# Gravity prior in human behaviour: a perceptual or semantic phenomenon?

**DOI:** 10.1007/s00221-020-05852-5

**Published:** 2020-06-21

**Authors:** Maria Gallagher, Agoston Torok, Johanna Klaas, Elisa Raffaella Ferrè

**Affiliations:** 1 Department of Psychology, Royal Holloway University of London, Egham, Surrey TW200EX UK; 2 School of Psychology, Cardiff University, Cardiff, UK; 3 Brain Imaging Centre RCNS, Magyar tudosok krt 2, Budapest, 1111 Hungary

**Keywords:** Graviception, Gravity prior, Perception, Visual motion

## Abstract

Humans show a gravitational advantage in perception: we are more precise at judging the speed of downwards-moving than upwards-moving objects, indicating that gravitational acceleration is an internalised prior. However, it is unclear whether this gravity prior is based on purely perceptual cues or whether it can incorporate semantic knowledge. Previous research has used only objects which are known to comply with gravity, possibly confounding semantic and perceptual cues. Here we have addressed this question by asking participants to judge the speed of objects that typically move coherently with gravity (ball) or against it (rocket). Our results showed a perceptual advantage for falling stimuli, irrespective of object identity, suggesting the gravity prior is based on perceptual cues.

## Introduction

Since the beginning of time, all living organisms have evolved under a constant terrestrial gravitational field of approximately 9.81 m/s^2^, known as 1 g. On Earth, gravity is always there; it is therefore not surprising that the physical constraints of Earth’s gravity are internalised in the human brain to shape our perception and action (Indovina et al. 2005). For instance, random accelerations are hardly perceived at all (Werkhoven et al. 1992), falling objects are expected to accelerate even when their velocity is constant (Zago et al. 2004), and observers misremember the location of moving objects in space (De Sá Teixeira 2016). In addition, gravity can influence eye movements, with improved smooth pursuit of objects which move according to 1 g vs objects which move according to weightlessness (0 g), reversed gravity (− 1 g), or hypergravity (2 g) (Delle Monache et al. 2015; Jörges and López-Moliner 2019). We are so exceptionally adapted to terrestrial gravity, that a *gravitational advantage* appears in perceptual judgements: observers are more precise in judging the speed of objects accelerating downwards compared to upwards (Bosco et al. 2008; Moscatelli and Lacquaniti 2011; Torok et al. 2019). The neural correlates of this gravitational advantage have been identified in a widespread brain network including the insular cortex, temporoparietal junction, premotor and supplementary motor areas, middle cingulate cortex, postcentral gyrus, thalamus and putamen (Indovina et al. 2005; Maffei et al. 2015).

The visual context seems to play a role in the gravitational advantage. For instance, while observers tend to anticipate the effects of gravity when intercepting objects, this is only the case when targets are embedded in a realistic visual scene (Miller et al. 2008). Accordingly, interception performance is similar under reversed and natural gravity conditions when targets are presented in a blank scene (Miller et al. 2008). Delle Monache et al. (2015) reported a key role of gravitational acceleration in guiding smooth pursuit and saccadic eye movements when target motion was embedded in a realistic context compared to a neutral background. Moreover, the gravitational advantage may depend on the gravity within the visual scene: when the environment is tilted relative to physical gravity, participants demonstrate an advantage for stimuli which move downwards according to the direction of the scene (Moscatelli and Lacquaniti 2011).

The gravitational advantage can be considered a proxy for the internalised gravity prior. Previous research has assumed that this prior is built from sensory experience of Earth gravity throughout the lifespan (Jörges and López-moliner 2017). However, it is not yet clear whether the gravity prior is purely made of constant exposure to online multimodal—vestibular, visual, proprioceptive, and visceral—gravitational signals, or whether it may also be built on *semantic knowledge* about physical gravitational constraints. Critically, in all previous studies (e.g., Moscatelli and Lacquaniti 2011; Torok et al. 2019; Zago et al. 2004), observers have been presented with objects which are most often seen to comply with the laws of gravity in the real world, such as a ball. Thus, it could be possible that the gravitational advantage was influenced by implicit semantic knowledge and expectations that a ball is normally falling down, rather than accelerating upwards.

Here we investigated whether participants would show the gravitational advantage when observing objects which move congruently with gravity and objects which can move against gravity. Participants judged the duration of motion for a ball or rocket moving downwards with or upwards against the terrestrial gravity vector in a virtual environment. A perceptual-based gravity prior predicts that the gravitational advantage would be present for both gravity-congruent and gravity-incongruent objects. However, a semantic-based gravity prior instead predicts that participants would show the gravitational advantage only for the ball, while performance would be similar in upwards and downwards conditions when viewing the rocket.

## Methods

### Participants

Twenty-four participants (four male, mean age = 20.25, SD = 1.67) were recruited from the Royal Holloway University subject pool. Seven participants were left-handed, while the remaining 17 participants were right-handed according to their Edinburgh Handedness Questionnaire (Oldfield 1971) results. Participants had no history of neurological, psychiatric, or vestibular disorders, and all had normal or corrected-to-normal vision. Written informed consent was obtained before commencing the experiment. The study received ethical approval from Royal Holloway University of London and was conducted in line with the Declaration of Helsinki.

### Stimuli and procedure

Before the experiment, participants received detailed instructions. Participants viewed a virtual environment on a liquid crystal display (LCD) computer monitor (LG Flatron, 17 inch, 60 Hz refresh rate), while seated with a chin rest 40 cm away from the screen. A cone was fitted to the screen to occlude additional cues from the external environment. The cone measured 30 cm in diameter at the participant end, and approximately 25 cm at the screen end. The centre of the cone was aligned vertically and horizontally to the centre of the screen.

**Fig. 1:**
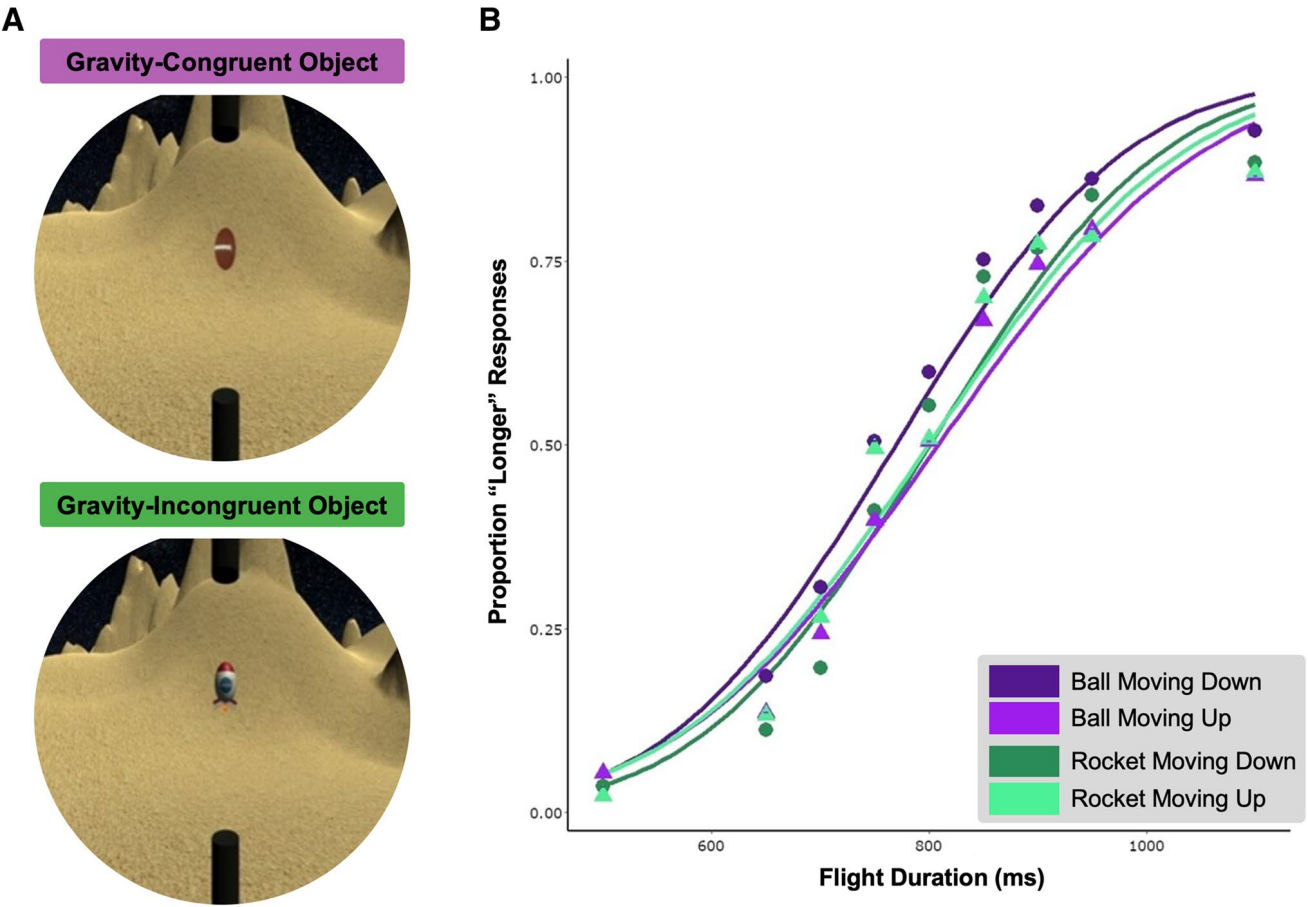
**a** Participants viewed a virtual environment depicting a planet. A rugby ball (top) or rocket (bottom) moved upwards against or downwards with gravity. **b** Average psychometric function for each object type and motion direction pooled across participants

The virtual environment was rendered in Unity 3D (2017.3.0f3, Unity Technologies 2018) and consisted of the surface of a planet with sand dunes and a night sky (Fig. 1a). The virtual environment measured 34 × 25.5 cm with 1024 × 768 resolution. Accordingly, participants saw approximately 56.62% of the virtual environment through the cone. A red dot (2 mm diameter) marked the centre of the environment and participants were asked to fixate on this point during the task. Two black tubes (1.5 cm diameter, 5 cm length) were placed in the sky and ground along the central midline, creating a path length of 15.5 cm. A rugby ball or rocket (both approximately 1.5 cm in length) accelerated upwards or downwards between the two black tubes (Fig. 1a). The magnitude of acceleration matched the drag of Earth gravity (9.81 m/s^2^).

We used a factorial design combining Motion Direction (Upward and Downward) and Object Type (Rocket and Ball) in four different blocks (i.e., Rocket moving Upwards; Rocket moving Downwards; Ball moving Upwards; Ball moving Downwards). Blocks were presented in a counterbalanced order across participants. Each block started with a learning phase in which participants were asked to memorise a reference speed of 3.57 m/s (duration = 800 ms; 60 reference trials per block, inter-stimulus interval (ISI) = 1300 ms). During the test phase, participants had to judge after each trial whether the object was moving faster or slower than the reference trials. Participants were instructed to press the left arrow on a keyboard if the object was moving faster and the right arrow if it was moving slower than the reference trials. The initial speed of the object during test trials was manipulated between 9.53 m/s and 0.05 m/s in nine steps resulting in nine different motion durations (0.5 s, 0.65 s, 0.7 s, 0.75 s, 0.80 s, 0.85 s, 0.90 s, 0.95 s, 1.10 s) as in previous studies (Moscatelli and Lacquaniti 2011; Torok et al. 2019). Each motion duration was presented 20 times, resulting in 180 test trials per block with ISI = 2300 ms. Thus, we used 2 motion directions × 2 object types × 9 motion durations × 20 repetitions for a total of 720 test trials across the whole experiment.

### Data analysis

Analyses were carried out in R software (R Core Team 2017) using lme4 (Bates et al. 2015) and MERpyschophysics (Moscatelli et al. 2012). Four participants were excluded from analysis as they showed poor performance (quantified by ${ \hbox{max} }_{P} - { \hbox{min} }_{P} < 0.5$, where *p* is the proportion of “slower” responses at the fastest (max) and slowest (min) test stimuli speeds). For each participant and condition, we computed the number of trials in which the test trial was considered slower than the reference, with slower coded as 1 and faster as 0. Missed responses, where the participant responded faster than 300 ms or slower than 2 s, were not included in the analysis (total = 3.21%).

The probability of a ‘slower’ response was calculated for each motion duration. Psychometric functions with probit link were constructed, based on previous studies (Moscatelli and Lacquaniti 2011; Torok et al. 2019):

$$\varPhi^{ - 1} \left[ {P\left( {y = 1} \right)} \right] = \beta_{0} + \beta_{1} x.$$

Precision was given by the $\beta_{0}$ parameter, while the point of subjective equality (PSE) was determined as

$${\text{PSE}} = - \frac{{\beta_{0} }}{{\beta_{1} }}.$$

The delta method (Casella and Berger 2002) was used to estimate the 95% confidence intervals for the point of subjective equality (PSE) for each subject. Discrimination thresholds, ${{\Delta }}T$, or just-noticeable differences (JND) were determined by

$$\Delta T = \frac{{T_{0.75} - T_{0.25} }}{2}$$

where $T_{0.25}$ and $T_{0.75}$ are the motion duration values matching the 0.25 and 0.75 probabilities of a “Slower” response. This $\Delta T$ was then used to calculate the Weber fraction:

$${\text{WF}} = \frac{\Delta T}{{T_{\text{standard}} }}$$

Both PSE and ${{\Delta }}T$ (JND) were fitted with General Linear Mixed Models (GLMM) to address the effect of motion direction (Downwards vs Upwards) and object type (Rocket vs Ball) on the population level. The GLMM included a single random intercept parameter, which was estimated for each subject and parameters for the fixed effects for the two object types, the two motion directions, the nine motion durations, and their interactions. For each parameter, we computed Wald statistics:

$$z = \frac{\beta }{\text{SE}}$$

where *β* is the estimated parameter and *SE* is respective standard error. The Slope parameters were normalised to the downwards motion’s slope.We also estimated the Bayes Factor (BF) from the Bayesian information criterion (BIC) of the null model and GLMM as

$${\text{BF}} = { \exp }\left( {\frac{{{\text{BIC}}_{\text{null}} - {\text{BIC}}_{\text{GLMM}} }}{2}} \right)$$

Conventional interpretations of the Bayes Factor were used, with values < 0.3 indicating moderate evidence for the null hypothesis, and values > 3 moderate evidence for the alternative hypothesis (Lee and Wagenmakers 2013).

## Results

Figure 1b shows the average psychometric function pooled across participants. Slopes for downwards motion are generally steeper than those for upwards motion across both object types, as predicted by the gravitational advantage (Moscatelli and Lacquaniti 2011).

JNDs were significantly lower in downwards vs upwards motion conditions (Wald *χ*^2^ = 9.62, *p* < 0.01) (Table 1). No significant difference in JND between object types (Wald *χ*^2^ = 0.15, *p* = 0.70), and no interaction (Wald *χ*^2^ = 0.68, *p* = 0.41) were found. The Bayes’ Factor was 0.05 (moderate evidence for the null hypothesis). These results suggest that object identity is not incorporated into the internal model of gravity.

**Table 1:** JND values (ms)

Object type	Motion direction	
	Upwards	Downwards
Ball	120.16 (6.27)	104.36 (5.00)
Rocket	115.52 (5.96)	106.67 (5.18)

Mean (SE)

PSEs were significantly different between downwards and upwards motion conditions (Wald *χ*^2^ = 32.34, *p* < 0.001), with lower PSEs for downwards vs upwards motion (Table 2). A significant difference was also found between object types (Wald *χ*^2^ = 34.19, *p* < 0.001), with lower PSEs for the ball vs rocket. A significant interaction between motion direction and object type was also found (Wald *χ*^2^ = 18.14, *p* < 0.001), with the lowest PSE for the rugby ball in the downwards motion condition.

**Table 2:** PSE values (ms)

Object type	Motion direction	
	Upwards	Downwards
Ball	796.48 (12.47)	766.14 (10.29)
Rocket	795.92 (12.04)	798.48 (11.21)

Mean (SE)

## Discussion

Gravity is a ubiquitous cue implicated in a range of human behaviours, such as object interception, verticality, and motion perception (Zago et al. 2004; de Rugy et al. 2012; Lacquaniti et al. 2015). A gravitational advantage has been reported, whereby individuals are more precise at judging the motion duration of objects which fall congruently with gravity (Moscatelli and Lacquaniti 2011; Torok et al. 2019). These findings suggest that observers use an internalised gravity prior when forming perceptual judgements. However, it is unclear whether the internalised gravity prior is based on purely perceptual information, or whether it also incorporates semantic knowledge regarding a particular object’s usual interaction with gravity. Here we investigated whether participants would exhibit the gravitational advantage for objects which typically comply with gravitational laws and those which move against gravity. The gravitational advantage was present for downwards motion conditions independently from object types. Thus, the gravity prior does not seem to be built on semantic knowledge about the physical constraints of gravity.

Our results suggest that the gravity prior is predominantly based on perceptual cues, rather than semantic knowledge regarding objects. A perceptual-based prior may incorporate knowledge that the gravity vector is typically aligned with the body axis, as the head is usually upright (Lacquaniti et al. 2015; Mittelstaedt 1983). For instance, individuals in a weightless environment, where a physical gravitational reference is absent, therefore revert to basing their perception of verticality on the location of the body axis (de Winkel et al. 2012). Importantly, cues from the context also seem crucial for anticipating the effects of gravity (Miller et al. 2008; Moscatelli and Lacquaniti 2011; Delle Monache et al. 2015). Accordingly, visual cues for the direction of verticality, such as the orientation of objects or the location of the sky and ground, may also play a key role in the gravity prior.

Here we found that the gravitational advantage was similar for both objects which can move against gravity and those which typically move with gravity, suggesting no influence of object identity, or semantics in general, on the precision of speed judgements. Curiously, however, we found a significant interaction between object type and movement direction on the point of subjective equality, with participants perceiving the downwards moving ball as faster than the other conditions. These findings may contrast with previously-reported results suggesting that upwards-moving stimuli may be perceived as faster than downwards-moving ones, particularly at higher speeds (Thompson and Stone 1997). However, differences between the stimuli and methods may account for this discrepancy: specifically, here we displayed objects moving within a scene, while previous studies have used simple gratings. Thus, the additional context provided by the virtual environment may have influenced participants’ speed judgements beyond what may be predicted by simple low-level visual features. Recently, Moscatelli et al. (2019) suggested that biases in perceived speed may be influenced by priors for motion dynamics within a scene, which may depend on factors such as gravity and the scene medium (i.e., water or air). Accordingly, downwards moving targets with high luminance contrast were perceived as faster than upwards moving and lower contrast targets (Moscatelli et al. 2019). Similarly, semantics concerning the object may also have influenced the expected motion dynamics of the scene, affecting speed biases independently from the gravitational advantage. Thus, the precision of speed judgements depends solely on a prior for gravity, resulting in a similar gravitational advantage for both gravity-congruent and incongruent objects. By contrast, biases in speed judgements may arise from broader scene dynamics, and may subsequently be affected by object identity. Consequently, a downwards moving ball is perceived as faster than an upwards moving one, while knowledge that rockets can be propelled upwards results in similar speed judgements in both upwards and downwards conditions.

To avoid discrepancies in visual saliency and to closely match previous studies (Moscatelli and Lacquaniti 2011; Torok et al. 2019), we presented both the rugby ball and rocket at the same size and scale within the virtual environment. It may be possible that the rocket condition was significantly less realistic than the rugby ball condition, considering that a real rocket would be many times larger than we presented here. A rocket was chosen to emphasize semantic differences. While this may have resulted in less realism for the rocket condition, people have clear semantic knowledge concerning the usual movement trajectories of a rocket compared to a ball. However, an open question remains whether differences in gravitational bias are present when objects are presented with greater realism (i.e., correct scaling within the virtual environment). It is also important to note that while rockets can move against gravity, they do not move at gravitational acceleration. Here, we ensured that the acceleration of the objects was identical in both upwards and downwards conditions to closely match previous studies of the gravitational advantage (Moscatelli and Lacquaniti 2011; Torok et al. 2019). Future studies might focus on whether the gravitational advantage would be modulated if the rocket was presented with a more realistic upwards acceleration profile.

 Evidence for the role of the gravity prior in perception is growing. Investigating which factors influence gravity-related perceptual judgements is therefore an expanding area of research. While previous studies have found that perception and action is more precise for objects obeying the laws of gravity, the role of object-related information has largely been neglected. Here we found that participants exhibited the same gravitational bias whether observing objects which typically obey gravitational laws or those which typically violate them. Thus, our findings suggest that the gravity prior is largely based on perceptual information, rather than semantic knowledge of the effect of gravity on objects.

## Data Availability

All data and materials are available from the authors on request.

## Code availability

All analysis and stimulus presentation codes are available from the authors on request.
